# The University of Maryland Dental Department

**Published:** 1889-09

**Authors:** 


### Editorial, Etc.

THE UNIVERSITY OF MARYLAND DENTAL DEPARTMENT.—This Dental Department is a member of the "National Association of Dental Faculties," and also holds permanent membership in "The American Dental Association." It is also on the list of Dental Schools whose diplomas are recommended by "The National Association of State Dental Examining Boards to all State Dental Examining Boards for recognition.

Examinations of Junior Students will be held at the close of each Regular or Winter Session, for admission to the Senior Class.

In accordance with a resolution passed by the "National Association of Dental Faculties," at the annual meeting held at Saratoga, Aug. 1889, and also adopted by the "American Dental Association" at its recent meeting, the rule requiring three sessions of five months each in separate years before graduation, will go into effect on and after October 1st, 1891; until that time the rule now in operation requiring two sessions of five months each in separate years, will continue.

The following officers were elected at the recent annual meeting of "The National Association of Dental Faculties:"

*President:* Prof. James Truman, Pennsylvania.
*Vice-President:* Prof. L. D. Carpenter, Georgia.
*Secretary:* Prof. Junius E. Cravens, Indiana.
*Treasurer:* Prof. A. W. Harlan, Illinois.
*Executive Committee:* Prof. Frank Abbott, New York.
Prof. J. Taft, Ohio.
Prof. F. J. S. Gorgas, Maryland.

An extensive addition to the Dental Infirmary and Laboratory Building of the University of Maryland is now being erected, and will be completed during the present month, August, 1889.

This addition will increase the length of the Dental Infirmary to almost one hundred feet, and add to the Laboratory 44 by 31 feet.

It will also afford an extensive Museum Hall, rendered necessary to accommodate the rapidly increasing collection of pathological and other specimens of which this Dental Museum is composed.

